# Efficacy of Fecal Microbiota Transplant on Behavioral and Gastrointestinal Symptoms in Pediatric Autism: A Systematic Review

**DOI:** 10.3390/microorganisms11030806

**Published:** 2023-03-22

**Authors:** Zahra Dossaji, Adam Khattak, Kyaw Min Tun, Mark Hsu, Kavita Batra, Annie S. Hong

**Affiliations:** 1 Department of Medical Education, Kirk Kerkorian School of Medicine at UNLV, University of Nevada, Las Vegas, NV 89102, USA; 2 Office of Research, Kirk Kerkorian School of Medicine at UNLV, University of Nevada, Las Vegas, NV 89102, USA; 3 Division of Gastroenterology and Hepatology, Department of Internal Medicine, Kirk Kerkorian School of Medicine at UNLV, University of Nevada, Las Vegas, NV 89102, USA

**Keywords:** fecal microbiota transplantation, pediatric, autism spectrum disorder, gut microbiome, microbiota, constipation, diarrhea, behavior

## Abstract

**Background and Aims:** There is a high prevalence of gastrointestinal-related (GI) symptoms among children with autism spectrum disorder (ASD), which is associated with the severity of behavioral symptoms. Fecal microbiota transplantation (FMT) is a proposed therapeutic strategy that aims to address the dysregulation of the gut microbiome among children with ASD. Our study performed the first systematic review aimed to evaluate the benefits of FMT on the behavioral and gastrointestinal symptoms of pediatric patients with autism. **Methods:** A literature search was performed using variations of the keywords “pediatrics” and “fecal microbiota transplantation” in PubMed, EMBASE, CINAHL, Cochrane, and Web of Science from inception to 30 June 2022. Four studies that met the eligibility criteria were included in the systematic review. The efficacy of FMT on behavioral symptoms was measured by the difference in Aberrant Behavior Checklist (ABC) and Child Autism Rating Scale (CARS) scores before and after FMT. **Results:** We found a statistically significant improvement (*p* < 0.05) in ABC and CARS scores following FMT, with a statistically significant decrease in scores observed across all studies. In addition, substantial improvements in gastrointestinal symptoms were observed across all studies. **Conclusion:** Our findings suggest that FMT may offer a promising intervention for treating both behavioral and gastrointestinal symptoms in pediatric patients with autism.

## 1. Introduction

Autism spectrum disorder (ASD) is a group of disorders characterized by varying degrees of difficulty in communication and social interaction, as well as the presence of repetitive and restrictive behaviors [1]. ASD is one of the most common and challenging neurodevelopmental disorders in children [2]. The number of children diagnosed with ASD has increased significantly in the past four decades, affecting approximately one in forty-four children globally [3]. Those diagnosed with ASD can manifest a wide range of physical, physiological, and psychiatric comorbidities [4]. Although the cause of ASD is still unknown, the most proposed causes are physiological and metabolic disorders involving immunity, oxidative stress, and mitochondrial dysfunction [5]. There is currently no pharmacological cure for ASD, and existing therapies include a multidisciplinary approach involving occupational, behavioral, speech, and play therapies [6].

While several studies have shown that dysregulation of neural connectivity is the proposed pathogenesis of ASD, many ASD children exhibit gastrointestinal (GI) symptoms, such as diarrhea, constipation, and abdominal pain, suggesting an association with the gut microbiome. The gut microbiome is responsible for producing most of the body’s neurotransmitters, and disruptions in the environment are thought to play a role in the development of neurological disorders. Previous studies have suggested that behavioral symptoms of ASD can be correlated with an imbalance of gut microbiota, deficiencies in serotonin signaling, disruptions and dysregulations in the “gut–brain axis” [7]. The gut–brain axis influences the emotional and cognitive centers of the brain via a bidirectional connection between the central nervous system and enteric nervous system to maintain gastrointestinal homeostasis [8]. Many studies have identified abnormalities in GI physiology in children with ASD, suggesting that changes in the intestinal flora may be related to ASD symptom severity [9].

There has been a growing interest in rebalancing the gut microbiota to treat ASD. While other reviews have analyzed various interventions with prebiotics, probiotics, vitamin A supplementation, and antibiotics administration, which have been shown to reduce GI and behavioral symptoms in individuals with ASD [10], there is limited data available regarding fecal microbiota transplantation (FMT) as a therapeutic modality for ASD in the pediatric population [11]. FMT is a non-pharmacological medical therapy in which fecal material from a donor is transferred into the recipient orally, endoscopically, or rectally. It is mostly used as a therapeutic strategy for antibiotic-resistant *Clostridium difficile* infections and has recently been recognized for its potential to treat other chronic inflammatory diseases, such as obesity and hepatic encephalopathy [12]. It has been demonstrated that following FMT, the ASD microbiome can result in a vastly different microbiome from baseline that closely resembles those of their donors and individuals without ASD [13].

Since altering the gut microbiota in childhood has been shown to be effective in reducing the severity of ASD, we aimed to examine and synthesize the currently available literature focused on the role or utility of FMT [14]; therefore, we performed the first systematic review of FMT as a potential therapy for behavioral and GI-related symptoms in pediatric patients with ASD.

## 2. Methods

### 2.1. Search Strategy

A comprehensive literature search was performed across five major databases (PubMed/Medline, Embase, CINAHL, Cochrane, and Web of Science) using keywords “fecal microbiota transplant” and “pediatric” to distinguish all studies published from inception through 30 June 2022. A total of 575 studies were identified for review.

Prior to screening the studies for eligibility, our review was registered on PROSPERO (PROSPERO registration number CRD42022343342). See Supplementary Materials for detailed search terms.

### 2.2. Eligibility Criteria

Studies which satisfied the following inclusion criteria: (1) involved administration of FMT in patients with ASD; (2) had pediatric patients under age 21 years with a diagnosis of ASD; (3) reported patient data and outcomes after fecal infusion; (4) involved patients of any sex; (5) included minimum follow-up of at least 1 month. The diagnosis of autism could be made by any recognized standardized score. In 2017, the American Academy of Pediatrics defined adolescence from 12 to 21 years of age and identified 21 years as the upper age limit of the pediatric population [15]. Furthermore, several FMT studies on pediatrics included age up to 21 years in the sample. Therefore, patients of age up to 21 years old were included in our study.

Exclusion criteria: (1) case reports with less than 5 patients; (2) published abstracts, letters to editor, and commentaries or reviews that did not include new patient data; (3) studies without patient data; (4) studies not published in English; (5) animal studies. Case series with more than 5 patients were included in our systematic review. The threshold for the number of patients that distinguished between case series (5 or more patients) and case reports (less than 5 patients) was derived from the paper by Abu-Zidan et al. [16].

### 2.3. Study Selection and Data Extraction

A total of 575 articles were identified in the initial search. Two authors (K.T. and M.H.) independently reviewed these titles and abstracts, after which seven articles were extracted from the initial abstract search for identifying the primary endpoints with relevant data. The full texts were then reviewed by two of the following authors (Z.D. and A.K.), after which four remaining studies fulfilled the eligibility criteria. In cases of disagreement, a senior reviewer (A.S.H.) reviewed the article and a final decision was made.

**Figure 1:**
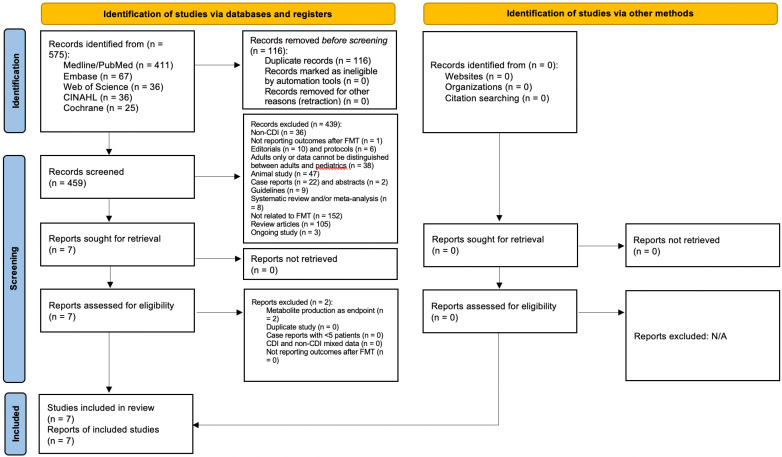
Preferred reporting items for systematic reviews and meta-analyses (PRISMA). (Reprinted with permission from Ref. [18].

The study selection process by preferred reporting items for systematic reviews and meta-analyses (PRISMA) statement is detailed in Figure 1 [17]. A summary of included studies is shown in Table 1. A summary of excluded studies is shown in Supplementary Table S3. IRB review was not required as all data were extracted from published literature and no direct patient intervention was executed.

### 2.4. Quality Assessment

The Newcastle–Ottawa Scale (NOS) was utilized to assess the methodological quality in observational studies such as the case-control and cohort studies [23]. The risk of bias was graded using a star system regarding subject selection, subject comparability, and exposure and outcome assessment. A study was deemed low risk of bias if it received a total of 8 to 9 stars, medium risk of bias if it received 6 to 7 stars and high risk of bias if it received less than 5 stars.

The risk of bias in non-randomized studies of interventions (ROBINS-I) was utilized to evaluate the risk of bias in non-randomized studies [24]. The scale includes seven domains of bias: two included in the pre-intervention stage, one at the intervention stage, and the last four in the post-intervention stage. If at least one of the domains was rated as high, the overall trial was considered at high risk of bias. If all the domains were rated as low, the trial was considered at low risk of bias.

The quality appraisal was performed by two authors (Z.D and A.K). If there was any disagreement, a senior reviewer (A.H.) evaluated the article and achieved consensus through discussion. See Supplementary Materials for the quality assessment scores for each study.

### 2.5. Study Outcomes

The primary endpoint of the study was the efficacy or success of FMT with improvement in behavioral symptoms. This was measured by the difference in scores in Aberrant Behavior Checklist (ABC) and Child Autism Rating Scale (CARS) scores before and after FMT, to see if there was a statistically significant improvement. We reported *p*-values from derived *t*-tests across the studies, and statistical significance was determined using a *p*-value of less than 0.05 [13]. ABC and CARS are assessment scales that are widely used in ASD research to evaluate the severity of ASD symptoms in children. The ABC consists of 58 items to assess problem behaviors in five domains common to children with ASD, such as irritability, lethargy, stereotypy, hyperactivity, and inappropriate speech [25,26]. A score of >53 points correlates to a high possibility of ASD. This questionnaire was filled out by the patient’s caregiver across the studies. CARS is a 15-item behavioral rating scale that can be used to diagnose ASD and categorize the overall severity of the symptoms, with a maximum score of 60 [27]. Scores of light-to-moderate are 30–36 and scores greater than 36 suggest severe symptoms [28]. This scale was completed by a qualified health professional across the studies.

The secondary endpoint of the study was the efficacy or clinical success of FMT with improvement in gastrointestinal symptoms. This was evaluated by the Bristol Stool Forming Scale (BSFS) and the Gastrointestinal Symptom Rating Scale (GSRS). The BSFS analyzes stool consistency by utilizing a picture scale as a reference to assess the morphological and trait characteristics of various stools (1 = very hard, 3–5 = normal fecal function, 7 = liquid) [29]. The BSFS was utilized across all the studies. The DSR (daily stool record) primarily included a rating of the stool utilizing the BSFS recorded daily with higher scores correlating with improvement. The GSRS is an assessment of GI symptoms filled out by the patient’s caregiver. It is based on 15 questions which are scored into 5 domains: abdominal pain, reflux, indigestion, diarrhea, and constipation [13].

## 3. Results

Through a systematic literature search, we found a total of four studies performing FMT on pediatric ASD patients, seen in Table 1. This included two retrospective cohort studies and two non-randomized, open-label clinical trials. A total of 149 pediatric patients were included in this study. All patients received a diagnosis of ASD by autism diagnostic interview-revised (ADI-R) and the fifth edition of diagnostic and statistical manual of mental disorders (DSM-5). In Kang’s study, 18 patients with moderate to severe gastrointestinal symptoms were recruited and compared to a control of neurotypical patients [22]. Similarly, Li recruited 40 patients with baseline GI symptoms [13]. Pan utilized a retrospective review of 42 patients with ASD who received FMT and reported data on GI symptom changes in patients with (n = 21) and without (n = 21) baseline constipation [20]. His study also assessed the optimal number of FMT courses required to see an improvement in behavioral symptoms by analyzing the difference in scores after each course [28]. Zhang’s study of 49 patients reported outcomes based on whether there was constipation by Rome-IV criteria (n = 24) or no baseline abnormal fecal form (n = 25) [21]. Kang and Li’s study patients were given only one complete course of FMT, while Zhang’s received two courses and Pan’s received up to five courses. Follow-up started as early as 2 weeks up to 8 weeks post infusion [13,27,28,29]. Recruited patients’ baseline characteristics are summarized in Table 2. Results of the primary and secondary endpoints are in Table 3 and Table 4 respectively. A summary of Pan’s results is shown in Table 5.

## 4. Discussion

For our primary efficacy endpoint of FMT on behavioral symptoms, we found that there was a statistically significant decrease in ABC and CARS scores across all studies when compared to baseline scores. Zhang′s study showed that ABC scores decreased in both constipation and non-constipation groups, but this benefit was not statistically significant until the second course in the baseline constipation group. Zhang′s study also demonstrated that CARS scores decreased after both initial and second courses, but the change in score was just below the cutoff for statistical significance in the baseline constipation group after the initial course. Both Kang and Li′s study used only one course of FMT, but they found significant improvements in both ABC and CARS scores. Kang later published a follow-up study on his initial patients, which showed that improvements persisted for two years after FMT concluded. Based on CARS, he reported that the severity of ASD at the two-year follow-up was 47% lower than the baseline compared to 23% lower at the end of treatment. At the beginning of the open-label trial, 83% of participants were categorized as having severe ASD according to CARS. In the same follow-up study, only 17% were rated as severe, 39% were in the mild to moderate range, and 44% of participants were below the ASD diagnostic cut-off scores [9].

For our secondary endpoint, which evaluated the efficacy of FMT on gastrointestinal symptoms, substantial improvements were observed across all studies. All studies found that FMT resulted in improved scores in BSFS and their respective subcategories of hard, soft/liquid, and no stool. Li′s study also revealed that there were no statistical differences between the oral or rectal FMT group, indicating that both routes of FMT improved stool characteristics in ASD children [13]. Kang showed an 80% reduction in GI symptoms at the end of treatment, including significant improvements in symptoms of constipation, diarrhea, indigestion, and abdominal pain, which also persisted 8 weeks after treatment [29]. Kang’s two-year follow-up study maintained a 58% reduction in their scores [9]. His two-year follow-up study also analyzed whether improvements in GI and ASD severity were significantly correlated and found that changes in ABC and CARS scores were positively correlated with changes in GSRS scores (Spearman correlation test, r > 0.7 and *p* < 0.005), implying a potential link between the severity of GI and behavioral symptoms.

It is well-established that children with ASD have a higher incidence of constipation and other GI symptoms, with reported rates of 22.2% and 46.8%, respectively [30]. Mazurek′s study found a 3.95% higher prevalence of anxiety in children with ASD who experienced chronic constipation compared to those without GI-related symptoms [31]. Zhang investigated the effectiveness of FMT in ASD patients without constipation or related GI symptoms and compared them to a group of ASD children with constipation. His study found a statistically significant improvement in ABC and CARS scores in the group without constipation, with no worsening of GI symptoms after treatment [20]. Further double-blinded placebo-controlled studies that include a control group of children with ASD without gastrointestinal symptoms would provide additional insights into whether FMT can improve the symptoms of children with ASD regardless of the presence of initial GI symptoms.

Our study had several limitations that should be taken into consideration when interpreting the results. Firstly, the lack of published data on FMT in ASD pediatric patients limited our ability to conduct a meta-analysis of randomized controlled trials. Additionally, the absence of raw data for adverse events prevented us from evaluating the safety of FMT in this population. While Zhang’s study mentioned the absence of a placebo control arm was due to ethical reasons, the other studies did not specify the reason for this absence [21]. This may have introduced bias in participant selection in the study. Secondly, the use of different scoring systems and variability in the ABC scales used among the studies made it challenging to combine data and perform a meta-analysis. Developing a standardized scoring system would be beneficial in future studies to enable data comparability and make more conclusive statements. Thirdly, the reduced number of studies identified in our search did not allow us to perform a publication bias analysis. Fourthly, the use of previously published data limited our ability to control for confounding factors, such as facility protocols for FMT administration and pre-FMT treatment. For example, some of the studies used washed microbiota transplant (WMT) preparations, which we used interchangeably with FMT, as the current literature has not shown a difference in efficacy [19,29]. Finally, the variability in the number of FMT courses and infusions among studies limited our ability to draw conclusions about the optimal number of FMT courses required for the maximum benefit [28,29]. Future studies with a larger number of participants and longer-term follow-ups are needed to address these limitations and provide more definitive conclusions regarding the efficacy and safety of FMT in ASD pediatric patients. Additionally, a larger sample size would enable a subgroup analysis to identify potential differences in treatment effects.

Overall, we conclude that although the available data is limited, FMT has shown potential significant benefits for both behavioral and gastrointestinal symptoms in pediatric patients with ASD. Alterations in the gut microbiome following fecal transplant of healthy donors and its potential effect on the gut–brain axis provide a possible explanation [8]. Multiple courses of FMT appear to maintain significant improvement compared to baseline, although, according to Pan’s study, the change in scores between each additional infusion loses significance after the third infusion. While acknowledging the limitations of the current data, FMT remains a promising novel therapy for ASD patients, and further randomized controlled studies are warranted.

## Figures and Tables

**Table 1:** A summary of the included studies. WMT* refers to washed microbiota transplant, which consists of an automatic washing process of the donor fecal suspension. WMT has not been found to have a difference in efficacy compared to standard FMT. Therefore, WMT and FMT are frequently assessed interchangeably [19].

Author/Year	Study Design	Pre-FMT Intervention	Intervention	Follow-Up
Pan 2022 [20]	Retrospective cohort study	None	WMT* injected through a transendoscopic enteral tube daily for 6 consecutive days. Participants received 2–5 treatments administered 4 weeks apart.	About 1 month after each treatment course
Zhang 2022 [21]	Retrospective cohort study	Probiotics (*Bifidobacteria* capsule, compound *Lactobacillus acidophilus* tablets, etc.) before and during FMT	WMT* injected via transendoscopic enteral tubing or nasojejunal tube daily for 6 days. Participants received two treatments administered 4 weeks apart.	8 weeks after each treatment course
Kang 2017 [22]	Open-label, non-randomized clinical trial	2-week antibiotic treatment with vancomycin, Prilosec, and Moviprep followed by a clear liquid diet the day before FMT	Initial high dose of FMT administered orally followed by lower oral dose daily for 8 weeks or one-time rectal dose of FMT followed by lower oral dose daily for 7 weeks.	8 weeks after treatment
Li 2021 [13]	Open-label, non-randomized clinical trial	2 L of polyethylene glycol	FMT administered orally via freeze-dried capsules or via colonoscopy once a week for 4 weeks.	0 weeks and 8 weeks after treatment

**Table 2:** Summary of patient population. NR = not reported.

Author/Year	Location (City, Country)	Population Characteristics	Sample Size (n)	Age (Mean)	Male (n)	Female (n)	BMI (Mean)	Baseline ABC Score (Mean)	Baseline CARS Score (Mean)	Baseline BSFS Score (Mean)
Pan 2022 [20]	Guangzhou, China	ASD children	42	6.00	34	8	17.01	59.00	36.00	3.00
Zhang 2022 [21]	Guangzhou, China	ASD children (24 with constipation, 25 without constipation)	49	5.67	41	8	NR	Constipation group: 56.21 Without constipation: 63.52	Constipation group: 35.25 Without constipation 36.64	Constipation group: 1.29 Without constipation 4.08
Kang 2017 [22]	Phoenix, United States of America	ASD children who had moderate to severe gastrointestinal problems	18	10.8	16	2	18.2	NR	NR	No stool: 33% (n = 5.94) Type I/II: 19% (n = 3.42) Type VI or VII: 10% (n = 1.8)
Li 2021 [13]	Nanjing, China	ASD children who had symptoms of the GI tract (constipation, diarrhea)	40	8.03	37	3	17.96	NR	NR	NR

**Table 3:** Efficacy of FMT in behavioral symptoms. All ABC and CARS scores reported at the end of first and additional treatment are reported in comparison to baseline scores prior to FMT. ABC and CARS scores reported at follow-up are reported in comparison to baseline scores prior to FMT. Not all studies provided data on the average scores of participants at baseline, after treatment or at follow-up. ** See Table 1. NR= Not reported. N/A= Not applicable.

Author/Year	ABC Score end of First Treatment	ABC Score End of Additional Treatment(s)	ABC Score at Follow-Up	CARS Score End of First Treatment	CARS Score End of Additional Treatment(s)	CARS Score at Follow-Up
Pan 2022 [20]	Decrease (*p* < 0.001)	2nd WMT **: Lower scores (*p* < 0.001) 3rd WMT: Lower scores (*p* < 0.001) 4th WMT: Lower scores (*p* < 0.01) 5th WMT: Lower scores (*p* < 0.05)	NR	Decrease (*p* < 0.0001)	2nd WMT: Lower scores (*p* < 0.0001) 3rd WMT: Lower scores (*p* < 0.001) 4th WMT: Lower scores (*p* < 0.01) 5th WMT: Lower scores (*p* < 0.05)	NR
Zhang 2022 [21]	Constipation group: Decrease; 50.92 (*p* = 0.286). Non-constipation group: Decrease; 57.56 (*p* = 0.309).	2nd WMT: Constipation group: Decrease; 46.54 (*p* = 0.046). Non-constipation group: Decrease; 52.88 (*p* = 0.053).	NR	Constipation group: Decrease; 33.15 (*p* = 0.059). Non-constipation group: Decrease; 34.54 (*p* = 0.033).	2nd WMT: Constipation group: Decrease; 32.50 (*p* = 0.015). Non-constipation group: Decrease; 33.88 (*p* = 0.002).	NR
Kang 2017 [22]	Decrease (*p* < 0.01).	N/A	Scores remained lower 8 eight weeks after treatment compared to baseline (*p* < 0.01).	Decrease (*p* < 0.001).	N/A	Scores remained lower 8 weeks after treatment (*p* < 0.001).
Li 2021 [13]	Decrease (*p* < 0.0001)	N/A	Scores remained lower 8 weeks after treatment (*p* < 0.01)	Decrease by 10% (*p* < 0.0001)	N/A	Scores remained lower by 6%, 8 weeks after treatment (*p* < 0.0001).

**Table 4:** Efficacy of FMT on gastrointestinal symptoms. ** See Table 1. NR= Not reported. N/A= Not applicable.

Author/Year	BSFS Scores	GSRS	Constipation Symptoms
Pan 2022 [20]	Lower scores after the 1st, 2nd, 3rd, and 4th WMT ** (*p* < 0.05) 18.92% improvement (*p* = 0.016) in the number of children with normal fecal form after the first treatment compared to baseline.	N/A	Reduced after each WMT when compared to baseline. 1st WMT: (*p* < 0.05); resolution of constipation in 23.8% of patients after 1st WMT: (*p* = 0.001). 2nd and 3rd WMT: (*p* < 0.01) 4th WMT: (*p* < 0.001) 5th WMT: (*p* < 0.05)
Zhang 2022 [21]	Constipation group: After 1st WMT: Increase; 2.33 (*p* = 0 < 0.001). After 2nd WMT: Increase; 2.92 (*p* < 0.001). Non-Constipation group: After 1st WMT: Decrease; 3.96 (*p* = 0.599). After 2nd WMT: Decrease; 4.00 (*p* = 0.337).	N/A	NR
Kang 2017 [22]	Reduction in the reported percent of days with: Hard stool (Type I or II) by 13% (*p* = 0.07) at end of treatment and 16% (*p* = 0.002) 8 weeks after treatment Soft liquid stool (Type VI or VII) by 8% (*p* = 0.03) at end of treatment and 7% (0.09) 8 weeks after treatment	Average score dropped 82% from the beginning to the end of treatment (*p* < 0.001) and remained 77% improved 8 weeks after treatment (*p* < 0.001).	Reduction in the reported percent of days with no stool by 7% (*p* = 0.29) at end of treatment and 8 weeks after treatment
Li 2021 [13]	60% of participants reporting hard stools (BSFS Type I/Type II) reduced to 10% at week 12 (*p* < 0.001).	Average scores decreased by 35% at the end of the 4-week treatment (*p* < 0.0001) and remained improved 8 weeks after treatment (*p* < 0.0001).	NR

**Table 5:** Summary of Pan’s results analyzing the effect of an increasing number of microbiota transplantation courses on changes in the ABC and CARS scales before and after each additional course. Δ ABC: ABC score after WMT minus ABC score at primary baseline; Δ CARS: CARS score after WMT minus CARS score at primary baseline. CARS scores gradually decreased with additional WMT courses, but there were no statistically significant differences between two adjacent courses. ** See Table 1. NR = Not reported.

WMT ** Course	Δ ABC Scores (mean)	Δ CARS Scores (mean)
Second vs. first	−6.50 vs. −5.00, *p* = 0.045	NR
Third vs. second	14.04 vs. 8.83, *p* = 0.022	NR
Fourth vs. third	13.57 vs. 11.57, *p* = 0.527	2.75 vs. 2.00, *p* = 0.930
Fifth vs. fourth	19.40 vs. 16.60, *p* = 0.351	3.92 vs. 1.58, *p* = 0.084

## Data Availability

Data supporting the statements can be found on PubMed, EMBASE, CINAHL, Web of Science, Cochrane and Google Scholar.

## Supplementary Materials

The following supporting information can be downloaded at: https://www.mdpi.com/article/10.3390/microorganisms11030806/s1, Table S1: Assessing Risk of Bias for Observational Cohort Studies with Newcastle-Ottawa Scale; Table S2: Assessing Quality of Nonrandomized Studies Using Risk of Bias in Non-Randomized Studies of Interventions (ROBINS-I) Scale; Table S3: Summary of Excluded Studies.
