# Supplementary material for: Efficacy of Fecal Microbiota Transplant on Behavioral and Gastrointestinal Symptoms in Pediatric Autism: A Systematic Review
Source: Microorganisms. 2023 Mar 22;11(3):806. doi: 10.3390/microorganisms11030806 (PMC10056604; doi:10.3390/microorganisms11030806)
Supplement: Supplementary file 1 [file microorganisms-11-00806-s001.zip › microorganisms-2294574-supplementary.pdf]

Supplemental Table S1: Assessing Risk of Bias for Observational Cohort Studies with Newcastle-Ottawa Scale

	**Pan 2022 [20]**	**Zhang 2022 [21]**
**Selection**		
1. Representativeness of the exposed cohort (Truly or somewhat)	*	*
2. Selection of the non-exposed cohort (drawn from the same community as the exposed cohort)	No	*
3. Ascertainment of exposure (secure record or structured interview)	*	*
4. Demonstration that outcome of interest was not present at start of study (yes)	*	*
**Comparability**		
5. Study controls for underlying GI issues (IBD, Celiac)	*	*
6. Study controls for underlying brain malformation	*	*
**Outcome**		
7. Assessment of outcome (independent blind assessment or record linkage)	*	*
8. Was follow-up long enough for outcomes to occur (yes)	*	*
9. Adequacy of follow up of cohorts (complete)	*	*
**Total ***	8	9
Score 7-9 high quality, 4-6 moderate risk of bias, 0-3 high risk of bias.	High	High

Supplemental Table S2: Assessing Quality of Nonrandomized Studies Using Risk of Bias in Non-Randomized Studies of Interventions (ROBINS-I) Scale

		**Kang 2017 [19]**	**Li 2021 [13]**
**Selection**			
Pre Intervention	Bias Due to Confounding	High	High
	Bias in Selection of Participants into the Study	High	High
At Intervention	Bias in Classification of Interventions	Low	Low
	Bias Due to Deviations from Intended Interventions	High	Low
Post Intervention	Bias Due to Missing Data	High	Low
	Bias in Measurement of Outcomes	Low	Low
	Bias in Selection of the Reported Result	High	High
	Overall Risk-of-Bias	High	High

Supplemental Table S3: Summary of Excluded Studies

Author, year	Title	Reason
	AASLD 2020 Abstracts	Abstract
	AASLD 2020 Abstracts	Abstract
Li 2019	Efficacy analysis of fecal microbiota transplantation in the treatment of 2010 patients with intestinal disorders	Adult
Ding 2019	Long-Term Safety and Efficacy of Fecal Microbiota Transplant in Active Ulcerative Colitis	Adult
Bloom 2022	Fecal microbiota transplant improves cognition in hepatic encephalopathy and its effect varies by donor and recipient	Adult
Mendelsohn 2022	Fecal Microbiota Transplantation Is Safe for Clostridiodies difficile Infection in Patients with Solid Tumors Undergoing Chemotherapy	Adult
Meighani 2020	Fecal Microbiota Transplantation for Clostridioides Difficile Infection in Patients with Chronic Liver Disease	Adult
Yang 2020	Potential role of intestinal microflora in disease progression among patients with different stages of Hepatitis B	Adult
Dai 2019	Rescue fecal microbiota transplantation for antibiotic-associated diarrhea in critically ill patients	Adult
Li 2022	Restoration of the gut microbiota is associated with a decreased risk of hepatic encephalopathy after TIPS	Adult
Torres 2018	Recurrent Relatively Resistant Salmonella infantis Infection in 2 Immunocompromised Hosts Cleared With Prolonged Antibiotics and Fecal Microbiota Transplantation	Adult
Mehta 2018	Preliminary experience with single fecal microbiota transplant for treatment of recurrent overt hepatic encephalopathy-A case series	Adult
Niccum 2018	Zinc Deficiency and the Recurrence of Clostridium difficile Infection after Fecal Microbiota Transplant: A Retrospective Cohort Study	Adult
Chin 2017	Fecal Microbiota Transplantation for Recurrent Clostridium difficile Infection in Patients With Inflammatory Bowel Disease: A Single-Center Experience	Adult
Furuya-Kanamori 2017	Upper Versus Lower Gastrointestinal Delivery for Transplantation of Fecal Microbiota in Recurrent or Refractory Clostridium difficile Infection: A Collaborative Analysis of Individual Patient Data From 14 Studies	Adult
Park 2017	Perceptions of fecal microbiota transplantation for Clostridium difficile infection: factors that predict acceptance	Adult
Youngster 2016	Oral, frozen fecal microbiota transplant (FMT) capsules for recurrent Clostridium difficile infection	Adult
Pakyz 2016	Fecal microbiota transplantation for recurrent Clostridium difficile infection: The patient experience	Adult
Gu 2016	Identification of key taxa that favor intestinal colonization of Clostridium difficile in an adult Chinese population	Adult
Wei 2015	Fecal microbiota transplantation restores dysbiosis in patients with methicillin resistant Staphylococcus aureus enterocolitis	Adult
Cui 2015	Fecal microbiota transplantation through mid-gut for refractory Crohn's disease: safety, feasibility, and efficacy trial results	Adult
Youngster 2014	Oral, capsulized, frozen fecal microbiota transplantation for relapsing Clostridium difficile infection	Adult
Youngster 2014	Fecal microbiota transplant for relapsing Clostridium difficile infection using a frozen inoculum from unrelated donors: a randomized, open-label, controlled pilot study	Adult
Rubin 2013	Fecal microbiome transplantation for recurrent Clostridium difficile infection: report on a case series	Adult
Okahara 2020	Matching between Donors and Ulcerative Colitis Patients Is Important for Long-Term Maintenance after Fecal Microbiota Transplantation	Adult
Li 2020	Fecal Microbiota Transplantation for Ulcerative Colitis: The Optimum Timing and Gut Microbiota as Predictors for Long-Term Clinical Outcomes	Adult

Pringle 2019	Patients With Cirrhosis Require More Fecal Microbiota Capsules to Cure Refractory and Recurrent Clostridium difficile Infections	Adult
Brittnacher 2016	GUTSS: An Alignment-Free Sequence Comparison Method for Use in Human Intestinal Microbiome and Fecal Microbiota Transplantation Analysis	Adult
Gama 2021	Immunologic biomarkers, morbidity and mortality among HIV patients hospitalised in a Tertiary Care Hospital in the Brazilian Amazon	Adult
Cheng 2021	Fecal Microbiota Transplantation Is Safe and Effective in Patients With Clostridioides difficile Infection and Cirrhosis	Adult
Kelly 2021	Fecal Microbiota Transplantation Is Highly Effective in Real-World Practice: Initial Results From the FMT National Registry	Adult
Burlow 2018	Impact of Amoxicillin-Clavulanate followed by Autologous Fecal Microbiota Transplantation on Fecal Microbiome Structure and Metabolic Potential	Adult
Tvede 2015	Rectal bacteriotherapy for recurrent Clostridium difficile-associated diarrhoea: Results from a case series of 55 patients in Denmark 2000-2012	Adult
Kump 2013	Alteration of intestinal dysbiosis by fecal microbiota transplantation does not induce remission in patients with chronic active ulcerative colitis	Adult
Cao 2017	Dysbiosis contributes to chronic constipation development via regulation of serotonin transporter in the intestine	Animal Study
Stevens 2022	The balance between protective and pathogenic immune responses to pneumonia in the neonatal lung is enforced by gut microbiota	Animal Study
Hui 2022	Donor-dependent fecal microbiota transplantation efficacy against necrotizing enterocolitis in preterm pigs	Animal Study
Pearson 2022	IgM-associated gut bacteria in obesity and type 2 diabetes in C57BL/6 mice and humans	Animal Study
Yan 2022	Intestinal toxicity of micro- and nano-particles of foodborne titanium dioxide in juvenile mice: Disorders of gut microbiota-host co-metabolites and intestinal barrier damage	Animal Study
Li 2022	Fecal transplantation can alleviate tic severity in a Tourette syndrome mouse model by modulating intestinal flora and promoting serotonin secretion	Animal Study
Brunse 2022	Fecal filtrate transplantation protects against necrotizing enterocolitis	Animal Study
Zhang 2022	Gut microbiota dysbiosis promotes age-related atrial fibrillation by lipopolysaccharide and glucose-induced activation of NLRP3-inflammasome	Animal Study
Yang 2022	Human Fecal Microbiota Transplantation Reduces the Susceptibility to Dextran Sulfate Sodium-Induced Germ-Free Mouse Colitis	Animal Study
Mu 2022	Seizure modulation by the gut microbiota and tryptophan-kynurenine metabolism in an animal model of infantile spasms	Animal Study
Kim 2022	Maternal gut bacteria drive intestinal inflammation in offspring with neurodevelopmental disorders by altering the chromatin landscape of CD4 + T cells	Animal Study
He 2021	Colonization of fecal microbiota from patients with neonatal necrotizing enterocolitis exacerbates intestinal injury in germfree mice subjected to necrotizing enterocolitis-induction protocol via alterations in butyrate and regulatory T cells	Animal Study
Lin 2021	Multiomics Study Reveals Enterococcus and Subdoligranulum Are Beneficial to Necrotizing Enterocolitis	Animal Study
Hiltunen 2021	Preterm infant meconium microbiota transplant induces growth failure, inflammatory activation, and metabolic disturbances in germ-free mice	Animal Study
Han 2021	Bifidobacterium infantis Maintains Genome Stability in Ulcerative Colitis via Regulating Anaphase-Promoting Complex Subunit 7	Animal Study
Wang 2021	Influences of non-IgE-mediated cow's milk protein allergy-associated gut microbial dysbiosis on regulatory T cell-mediated intestinal immune tolerance and homeostasis	Animal Study
Gozalbo-Rovira 2021	Microbiota Depletion Promotes Human Rotavirus Replication in an Adult Mouse Model	Animal Study
Liu 2021	Gut microbiota mediates cognitive impairment in young mice after multiple neonatal exposures to sevoflurane	Animal Study
Yu 2021	β-Sitosterol Ameliorates Endometrium Receptivity in PCOS-Like Mice: The Mediation of Gut Microbiota	Animal Study
Li 2021	Icariin enhances youth-like features by attenuating the declined gut microbiota in the aged mice	Animal Study

Xi 2021	Microbiome-metabolomic analyses of the impacts of dietary stachyose on fecal microbiota and metabolites in infants intestinal microbiota-associated mice	Animal Study
Xiao 2021	Fecal Microbiome Transplantation from Children with Autism Spectrum Disorder Modulates Tryptophan and Serotonergic Synapse Metabolism and Induces Altered Behaviors in Germ-Free Mice	Animal Study
Qi 2021	A Novel and Reliable Rat Model of Autism	Animal Study
Amenyogbe 2021	Biogeography of the Relationship between the Child Gut Microbiome and Innate Immune System	Animal Study
Brunse 2021	Enteral broad-spectrum antibiotics antagonize the effect of fecal microbiota transplantation in preterm pigs	Animal Study
Zhao 2020	Restraining the TiO 2 nanoparticles-induced intestinal inflammation mediated by gut microbiota in juvenile rats via ingestion of Lactobacillus rhamnosus GG	Animal Study
Srivastava 2020	Reduced rotavirus vaccine efficacy in protein malnourished human-faecal-microbiota-transplanted gnotobiotic pig model is in part attributed to the gut microbiota	Animal Study
Goo 2020	The effect of fecal microbiota transplantation on autistic-like behaviors in Fmr1 KO mice	Animal Study
Ross 2020	Feasibility of fecal microbiota transplantation via oral gavage to safely alter gut microbiome composition in marmosets	Animal Study
Xi 2020	Stachyose increases intestinal barrier through Akkermansia muciniphila and reduces gut inflammation in germ-free mice after human fecal transplantation	Animal Study
DÁmato 2020	Faecal microbiota transplant from aged donor mice affects spatial learning and memory via modulating hippocampal synaptic plasticity- and neurotransmission-related proteins in young recipients	Animal Study
Lee 2020	Gut Microbiota-Derived Short-Chain Fatty Acids Promote Poststroke Recovery in Aged Mice	Animal Study
Liu 2020	Fecal microbiota transplantation by enema reduces intestinal injury in experimental necrotizing enterocolitis	Animal Study
Avila 2020	Protective effects of fecal microbiota transplantation in sepsis are independent of the modulation of the intestinal flora	Animal Study
McKinney 2020	The fecal microbiota of healthy donor horses and geriatric recipients undergoing fecal microbial transplantation for the treatment of diarrhea	Animal Study
Michael 2020	Malnutrition Decreases Antibody Secreting Cell Numbers Induced by an Oral Attenuated Human Rotavirus Vaccine in a Human Infant Fecal Microbiota Transplanted Gnotobiotic Pig Model	Animal Study
Torres 2020	Infants born to mothers with IBD present with altered gut microbiome that transfers abnormalities of the adaptive immune system to germ-free mice	Animal Study
Lei 2019	Enhanced GII.4 human norovirus infection in gnotobiotic pigs transplanted with a human gut microbiota	Animal Study
Zachariassen 2019	Cesarean section increases sensitivity to oxazolone-induced colitis in C57BL/6 mice	Animal Study
Dhakal 2019	Amish (Rural) vs. non-Amish (Urban) Infant Fecal Microbiotas Are Highly Diverse and Their Transplantation Lead to Differences in Mucosal Immune Maturation in a Humanized Germfree Piglet Model	Animal Study
Abdel-Gadir 2019	Microbiota therapy acts via a regulatory T cell MyD88/RORγt pathway to suppress food allergy	Animal Study
Chadchan 2019	Antibiotic therapy with metronidazole reduces endometriosis disease progression in mice: a potential role for gut microbiota	Animal Study
Spychala 2018	Age-related changes in the gut microbiota influence systemic inflammation and stroke outcome	Animal Study
Zhang 2017	Environmental spread of microbes impacts the development of metabolic phenotypes in mice transplanted with microbial communities from humans	Animal Study
Planer 2016	Development of the gut microbiota and mucosal IgA responses in twins and gnotobiotic mice	Animal Study
Nagy-Szakal 2015	Loss of n-6 fatty acid induced pediatric obesity protects against acute murine colitis	Animal Study
Brown 2016	Prolonged antibiotic treatment induces a diabetogenic intestinal microbiome that accelerates diabetes in NOD mice	Animal Study

Cassir 2016	Gut microbiota and the pathogenesis of necrotizing enterocolitis in preterm neonates	Animal Study
Spinner 2020	Fecal microbiota transplantation in a toddler after heart transplant was a safe and effective treatment for recurrent Clostridiodes difficile infection: A case report	Case report
Barfuss 2018	Cardiac allograft vasculopathy following fecal microbiota transplantation for recurrent C. difficile infection	Case report
Wu 2021	Fecal microbiota transplantation before hematopoietic stem cell transplantation in a pediatric case of chronic diarrhea with a FOXP3 mutation	Case report
Zhang 2021	Longitudinal dynamics of gut bacteriome, mycobiome and virome after fecal microbiota transplantation in graft-versus-host disease	Case report
Singh 2019	Oral fecal microbiota transplant for recurrent Clostridium difficile in pediatric autoimmune enteropathy	Case report
Stenberg 2022	Faecal transplantation in a two-year-old child with therapy-resistant Clostridiodes difficileinfection	Case report
Sabus 2021	Fecal Microbiota Transplantation for Treatment of Severe Clostridioides difficile Colitis in a Pediatric Patient With Non-Hodgkin Lymphoma	Case report
Menendez 2019	Severe neonatal anemia due to fetomaternal hemorrhage: an ilustrative case	Case report
Bulik-Sullivan 2018	Intestinal Microbial and Metabolic Alterations Following Successful Fecal Microbiota Transplant for D-Lactic Acidosis	case report
Sierra Salinas 2018	Faecal microbiota transplant in a child with very early onset inflammatory bowel disease	case report
Dow 2018	Clostridium difficile cure with fecal microbiota transplantation in a child with Pompe disease: a case report	case report
Davidovics 2017	Fecal Transplantation Successfully Treats Recurrent D-Lactic Acidosis in a Child With Short Bowel Syndrome	case report
Flannigan 2017	Changes in Composition of the Gut Bacterial Microbiome after Fecal Microbiota Transplantation for Recurrent Clostridium difficile Infection in a Pediatric Heart Transplant Patient	case report
Wang 2017	16S rDNA Gene Sequencing Analysis in Functional Dyspepsia Treated With Fecal Microbiota Transplantation	case report
Zhao 2017	The Effect of Fecal Microbiota Transplantation on a Child with Tourette Syndrome	case report
Loke 2016	Fecal microbial transplantation in a pediatric case of recurrent Clostridium difficile infection and specific antibody deficiency	case report
Kumagai 2016	Failure of Fecal Microbiota Transplantation in a Three-Year-Old Child with Severe Refractory Ulcerative Colitis	case report
Shimizu 2016	Repeated fecal microbiota transplantation in a child with ulcerative colitis	case report
Samuel 2016	NURSING ASSESSMENT FOR "DO IT YOURSELF" FECAL MICROBIOTA TRANSPLANTATION	case report
Vandenplas 2015	Fecal Microbial Transplantation in Early-Onset Colitis: Caution Advised	Case report
Wang 2015	Pediatric severe pseudomembranous enteritis treated with fecal microbiota transplantation in a 13-month-old infant	case report
Kahn 2012	Colonoscopic fecal microbiota transplant for recurrent Clostridium difficile infection in a child	case report
Russell 2010	Fecal bacteriotherapy for relapsing Clostridium difficile infection in a child: a proposed treatment protocol	case report
Ge 2017	Atypical Late-Onset Immune Dysregulation, Polyendocrinopathy, Enteropathy, X-Linked Syndrome with Intractable Diarrhea: A Case Report	case report
Beurden 2019	serial Microbiota Analysis after Fecal Microbiota Transplantation in a Child with Down's Syndrome	case report
Ooijevaar 2019	Faecal transplants for children with recurrent infections	Case series
Barfield 2018	Going to the Bank: Fecal Microbiota Transplantation in Pediatrics	case series
Bluestone 2018	Fecal Microbiota Transplantation for Recurrent Clostridium difficile Infections in Pediatric Hematopoietic Stem Cell Transplant Recipients	Case series

Kronman 2015	Fecal microbiota transplantation via nasogastric tube for recurrent clostridium difficile infection in pediatric patients	case series
Walia 2014	Efficacy of fecal microbiota transplantation in 2 children with recurrent Clostridium difficile infection and its impact on their growth and gut microbiome	case series
Pierog 2014	Fecal microbiota transplantation in children with recurrent Clostridium difficile infection	case series
Russell 2014	Fecal transplant for recurrent Clostridium difficile infection in children with and without inflammatory bowel disease	case series
Petito 2019	Commentary to "Safety, Clinical Response, and Microbiome Findings Following Fecal Microbiota Transplant in Children With Inflammatory Bowel Disease"	Editorial
Lynch 2015	Fecal Microbiota Transplantation for Recurrent Clostridium difficile Infection in Pediatric Patients: Encouragement Wrapped in Caution	Editorial
Kang 2019	Long-term benefit of Microbiota Transfer Therapy on autism symptoms and gut microbiota	Duplicate Study
Kattner 2022	About gladiators and a sacred disease	Editorial
Helve 2021	Giving faecal transplants to infants born by Caesarean section produced similar gut microbiotica results to vaginal deliveries	Editorial
Haifer 2020	Faecal microbiota transplantation as an elixir of youth	Editorial
Groves 2020	Winning with poo? Fecal microbiome transplantation as an emerging strategy for the management of recurrent Clostridioides difficile infection in children	Editorial
Russell 2016	New treatments for ulcerative colitis: do we have pediatric data?	Editorial
Goyal 2019	Author's Reply to Drs. Franco Scaldaferri and Valentina Petito, PhD	Editorial
Kociolek 2019	Response to: Treatment of (Recurrent) Clostridioides difficile Infections in Children and Adults	Editorial
Hourigan 2021	Updates and Challenges in Fecal Microbiota Transplantation for Clostridioides difficile Infection in Children	Guidelines
No author 2021	Clostridioides difficile infection: antimicrobial prescribing	guidelines
Rogers 2020	Clinical Guideline Highlights for the Hospitalist: Clostridium difficile Infections in Children	guidelines
Diorio 2018	Guideline for the Management of Clostridium Difficile Infection in Children and Adolescents With Cancer and Pediatric Hematopoietic Stem-Cell Transplantation Recipients	Guidelines
Davidovics 2019	Fecal Microbiota Transplantation for Recurrent Clostridium difficile Infection and Other Conditions in Children: A Joint Position Paper From the North American Society for Pediatric Gastroenterology, Hepatology, and Nutrition and the European Society for Pediatric Gastroenterology, Hepatology, and Nutrition	guidelines
Trubiano 2016	Australasian Society of Infectious Diseases updated guidelines for the management of Clostridium difficile infection in adults and children in Australia and New Zealand	guidelines
Alonso 2022	American Society for Transplantation and Cellular Therapy Series: #5-Management of Clostridioides difficile Infection in Hematopoietic Cell Transplant Recipients	Guidelines
Turner 2018	Management of Paediatric Ulcerative Colitis, Part 1: Ambulatory Care-An Evidence-based Guideline From European Crohn's and Colitis Organization and European Society of Paediatric Gastroenterology, Hepatology and Nutrition	Guidelines
Turner 2018	Management of Paediatric Ulcerative Colitis, Part 2: Acute Severe Colitis-An Evidence-based Consensus Guideline From the European Crohn's and Colitis Organization and the European Society of Paediatric Gastroenterology, Hepatology and Nutrition	guidelines
Tang 2017	Is frozen fecal microbiota transplantation as effective as fresh fecal microbiota transplantation in patients with recurrent or refractory Clostridium difficile infection: A meta-analysis?	Meta-analysis
Imdad 2018	Fecal transplantation for treatment of inflammatory bowel disease	Meta-analysis
Uzan-Yulzari 2021	Neonatal antibiotic exposure impairs child growth during the first six years of life by perturbing intestinal microbial colonization	Non ASD

Chehoud 2016	Transfer of Viral Communities between Human Individuals during Fecal Microbiota Transplantation	Non ASD
Korpela 2020	Maternal Fecal Microbiota Transplantation in Cesarean-Born Infants Rapidly Restores Normal Gut Microbial Development: A Proof-of-Concept Study	Non ASD
Merli 2020	Decolonization of multi-drug resistant bacteria by fecal microbiota transplantation in five pediatric patients before allogeneic hematopoietic stem cell transplantation: gut microbiota profiling, infectious and clinical outcomes	Non ASD
Karolewska-Bochenek 2018	A Two-Week Fecal Microbiota Transplantation Course in Pediatric Patients with Inflammatory Bowel Disease	Non ASD
Zhong 2019	Fecal microbiota transplantation for refractory diarrhea in immunocompromised diseases: a pediatric case report	Non ASD
Zhao 2020	The Efficacy of Fecal Microbiota Transplantation for Children With Tourette Syndrome: A Preliminary Study	Non ASD
Quagliariello 2020	Fecal Microbiota Transplant in Two Ulcerative Colitis Pediatric Cases: Gut Microbiota and Clinical Course Correlations	Non ASD
Yodoshi 2018	Fecal Microbiota Transplantation to Patients with Refractory Very Early Onset Ulcerative Colitis	Non ASD
Kellermayer 2015	Serial fecal microbiota transplantation alters mucosal gene expression in pediatric ulcerative colitis	Non ASD
Suskind 2015	Fecal microbial transplant via nasogastric tube for active pediatric ulcerative colitis	Non ASD
Guchte 2021	Dynamic Properties of the Intestinal Ecosystem Call for Combination Therapies, Targeting Inflammation and Microbiota, in Ulcerative Colitis	Non ASD
Karolewska-Bochenek 2021	Faecal Microbiota Transfer - a new concept for treating cytomegalovirus colitis in children with ulcerative colitis	Non ASD
Zhong 2021	Colonic Transendoscopic Enteral Tubing: Route for a Novel, Safe, and Convenient Delivery of Washed Microbiota Transplantation in Children	Non ASD
Suskind 2015	Fecal microbial transplant effect on clinical outcomes and fecal microbiome in active Crohn's disease	Non ASD
Goloshchapov 2020	Fecal microbiota transplantation for graft-versus-host disease in children and adults: methods, clinical effects, safety	Non ASD
Goyal 2018	Safety, Clinical Response, and Microbiome Findings Following Fecal Microbiota Transplant in Children With Inflammatory Bowel Disease	Non ASD
Nusbaum 2018	Gut microbial and metabolomic profiles after fecal microbiota transplantation in pediatric ulcerative colitis patients	Non ASD
Kunde 2013	Safety, tolerability, and clinical response after fecal transplantation in children and young adults with ulcerative colitis	Non ASD
Chen 2022	FTACMT study protocol: a multicentre, double-blind, randomised, placebo-controlled trial of faecal microbiota transplantation for autism spectrum disorder	Non ASD
Wilson 2021	Strain engraftment competition and functional augmentation in a multi-donor fecal microbiota transplantation trial for obesity	Non ASD
Leong 2020	Effects of Fecal Microbiome Transfer in Adolescents With Obesity: The Gut Bugs Randomized Controlled Trial	Non ASD
Xi 2019	Fecal microbiota transplantation in children does not significantly alter body mass index	Non ASD
Popov 2021	Pediatric Patient and Parent Perceptions of Fecal Microbiota Transplantation for the Treatment of Ulcerative Colitis	Non ASD
Pai 2021	Results of the First Pilot Randomized Controlled Trial of Fecal Microbiota Transplant In Pediatric Ulcerative Colitis: Lessons, Limitations, and Future Prospects	Non ASD
Liu 2017	Fecal microbiota transplantation induces remission of infantile allergic colitis through gut microbiota re-establishment	Non ASD
Cho 2019	Fecal Microbiota Transplant for Recurrent Clostridium difficile Infection in Pediatric Inflammatory Bowel Disease	Non ASD
Cui 2015	Step-up fecal microbiota transplantation strategy: a pilot study for steroid-dependent ulcerative colitis	Non ASD
Qureshi 2020	Not reporting ASD outcomes after FMT	Non ASD

Li 2020	Composition of "gold juice" using an ancient method based on intestinal microecology	Non ASD
Kelly 2014	Fecal microbiota transplant for treatment of Clostridium difficile infection in immunocompromised patients	Non ASD
Pai, N; Popov, J; Hartung, E; Hill, L; Thabane, L; Lee, C; Surette, M; Godin, D; Grzywacz, SJ; Moayyedi, P	RESULTS OF THE FIRST PILOT RANDOMIZED CONTROLLED TRIAL OF FAECAL MICROBIOTA TRANSPLANT FOR PEDIATRIC ULCERATIVE COLITIS	Non ASD
Popov, J; Hartung, E; Hill, L; Chauhan, U; Pai, N	FECAL MICROBIOTA TRANSPLANTATION: PERCEPTIONS AND EXPERIENCES IN A PEDIATRIC ULCERATIVE COLITIS POPULATION (PEDIFETCH TRIAL)	Non ASD
Pai, N; Popov, J; Hartung, E; Hill, L; Thabane, L; Lee, C; Surette, M; Godin, D; Grzywacz, K; Moayyedi, P	Results of the first pilot randomized controlled trial of faecal microbiota transplant for pediatric ulcerative colitis	Non ASD
Drewes 2019	Transmission and clearance of potential procarcinogenic bacteria during fecal microbiota transplantation for recurrent Clostridioides difficile	Non ASD
Hourigan 2019	Fecal Transplant in Children With Clostridioides difficile Gives Sustained Reduction in Antimicrobial Resistance and Potential Pathogen Burden	Non ASD
Li 2018	Clinical Efficacy and Microbiome Changes Following Fecal Microbiota Transplantation in Children With Recurrent Clostridium Difficile Infection	Non ASD
Barnes 2018	Competitively Selected Donor Fecal Microbiota Transplantation: Butyrate Concentration and Diversity as Measures of Donor Quality	Non ASD
Fareed 2018	Applying fecal microbiota transplantation (FMT) to treat recurrent Clostridium difficile infections (rCDI) in children	Non ASD
Hourigan 2015	Microbiome changes associated with sustained eradication of Clostridium difficile after single faecal microbiota transplantation in children with and without inflammatory bowel disease	Non ASD
Shimizu 2019	P043 OUTCOME OF THE REPETITIVE FECAL MICROBIOTA TRANSPLANTATION USING FECAL SOLUTION PREPARED UNDER THE ANAEROBIC CONDITION FOLLOWING THE ANTIBIOTIC PRETREATMENT IN EIGHT CHILDREN WITH ULCERATIVE COLITIS	Non ASD
Nicholson 2022	Efficacy and Outcomes of Faecal Microbiota Transplantation for Recurrent Clostridioides difficile Infection in Children with Inflammatory Bowel Disease	Non ASD
Li 2022	Characteristics and management of children with Clostridiodes difficile infection at a tertiary pediatric hospital in China	Non ASD
Ruan 2021	Alternative Diagnoses in Pediatric Fecal Microbiota Transplant Referral Patients	Non ASD
Nicholson 2020	Efficacy of Fecal Microbiota Transplantation for Clostridium difficile Infection in Children	Non ASD
Aldrich 2019	Analysis of Treatment Outcomes for Recurrent Clostridium difficile Infections and Fecal Microbiota Transplantation in a Pediatric Hospital	Non ASD
Zhang 2018	Safety of fecal microbiota transplantation in Chinese children: A single-center retrospective study	Non ASD
Brumbaugh 2018	An Intragastric Fecal Microbiota Transplantation Program for Treatment of Recurrent Clostridium difficile in Children is Efficacious, Safe, and Inexpensive	Non ASD
Buonsenso 2022	Clostridioides difficile Infection in Children: A 5-Year Multicenter Retrospective Study	Non ASD

McIlroy, J; Nalagatla, N; Hansen, R; Hart, A; Hold, G	FMT as a treatment for IBD: a national survey of gastroenterologists in the UK	Non ASD
Siranosian 2020	Author Correction: Acquisition, transmission and strain diversity of human gut-colonizing crAss-like phages	Not FMT
Huang 2021	Higher fibrotic content of endometriotic lesions is associated with diminished prostaglandin E2 signaling	Not FMT
Zhou 2021	Nanoarmour-shielded single-cell factory for bacteriotherapy of Parkinson's disease	Not FMT
Vallianou 2021	Understanding the Role of the Gut Microbiome and Microbial Metabolites in Non-Alcoholic Fatty Liver Disease: Current Evidence and Perspectives	Not FMT
Philips 2022	The role of gut microbiota in clinical complications, disease severity, and treatment response in severe alcoholic hepatitis	not FMT
Hathibelagal 2021	Evaluation of photoreceptor function in inherited retinal diseases using rod- and cone-enhanced flicker stimuli	not FMT
Gregory 2022	MetaPop: a pipeline for macro- and microdiversity analyses and visualization of microbial and viral metagenome-derived populations	Not FMT
Wilson 2021	Oral administration of maternal vaginal microbes at birth to restore gut microbiome development in infants born by caesarean section: A pilot randomised placebo-controlled trial	not FMT
Galipeau 2021	Novel Fecal Biomarkers That Precede Clinical Diagnosis of Ulcerative Colitis	not FMT
Alghamdi 2022	Bee Pollen and Probiotics May Alter Brain Neuropeptide Levels in a Rodent Model of Autism Spectrum Disorders	not FMT
Onuki 2021	Dopaminergic restoration of prefrontal cortico-putaminal network in gene therapy for aromatic l-amino acid decarboxylase deficiency	not FMT
Pinheiro 2021	Cryptococcal meningitis in non-HIV patients in the State of Amazonas, Northern Brazil	not FMT
Sasaki 2021	Growth stimulation of Bifidobacterium from human colon using daikenchuto in an in vitro model of human intestinal microbiota	not FMT
Warda 2021	A postbiotic consisting of heat-treated lactobacilli has a bifidogenic effect in pure culture and in human fermented faecal communities	not FMT
Rahne 2021	A retrospective European multicenter analysis of the functional outcomes after active middle ear implant surgery using the third generation vibroplasty couplers	not FMT
Bondue 2020	Effect of Bifidobacterium crudilactis and 3'-sialyllactose on the toddler microbiota using the SHIME® model	not FMT
Zhao 2020	Long-term Outcomes of Clip Coupler Implantation in Patients with Unilateral Congenital Aural Atresia	not FMT
Krol 2020	Mastoid Obliteration with S53P4 Bioactive Glass Can Make Bonebridge Implantation Feasible: A Case Report	not FMT
Troisi 2020	Genome, Environment, Microbiome and Metabolome in Autism (GEMMA) Study Design: Biomarkers Identification for Precision Treatment and Primary Prevention of Autism Spectrum Disorders by an Integrated Multi-Omics Systems Biology Approach	not FMT
Koo 2020	Strain Tracking to Identify Individualized Patterns of Microbial Strain Stability in the Developing Infant Gut Ecosystem	not FMT
Woodworth 2020	mSphere of Influence: Microbiome-Associated Phenotypes Are Modifiable	not FMT
Little 2020	Gut microbiome in primary sclerosing cholangitis: A review	not FMT
Manti 2020	Bacteriotherapy with Streptococcus salivarius 24SMB and Streptococcus oralis 89a nasal spray for treatment of upper respiratory tract infections in children: a pilot study on short-term efficacy	Not FMT
Tarantino 2020	Oral bacteriotherapy in children with recurrent respiratory infections: a real-life study	not FMT
Albuhairi 2020	Novel Therapies for Treatment of Food Allergy	not FMT

Song 2020	The Microbiota in Hematologic Malignancies	not FMT
Chen 2020	Probiotic mixtures with aerobic constituent promoted the recovery of multi-barriers in DSS-induced chronic colitis	not FMT
Walker 2019	Transcriptomic changes during TGF-β-mediated differentiation of airway fibroblasts to myofibroblasts	not FMT
Kılınç 2019	Prevalence, aetiology, and treatment of molar incisor hypomineralization in children living in Izmir City (Turkey)	not FMT
Figueiredo 2019	Human parvovirus B19 genotype 1 in suspected dengue patients of Tefé, Amazonas State, Brazil	not FMT
Araki 2019	Foveal avascular zone and macular vessel density after correction for magnification error in unilateral amblyopia using optical coherence tomography angiography	Not FMT
Morffy 2019	Composition of the gut microbiota transcends genetic determinants of malaria infection severity and influences pregnancy outcome	not FMT
Borrelli 2019	MACULAR MICROVASCULAR NETWORKS IN HEALTHY PEDIATRIC SUBJECTS	not FMT
Garcia-Fernandez 2019	Whole-genome sequencing reveals nosocomial Clostridioides difficile transmission and a previously unsuspected epidemic scenario	not FMT
Bauman 2019	Preliminary evidence of increased striatal dopamine in a nonhuman primate model of maternal immune activation	not FMT
Biagi 2019	Early gut microbiota signature of aGvHD in children given allogeneic hematopoietic cell transplantation for hematological disorders	not FMT
Tarantino 2019	Bacteriotherapy in children with recurrent upper respiratory tract infections	not FMT
Bellussi 2019	An overview on upper respiratory tract infections and bacteriotherapy as innovative therapeutic strategy	not FMT
La Mantia 2019	The role of bacteriotherapy in the prevention of adenoidectomy	not FMT
Passali 2019	The efficacy and tolerability of Streptococcus salivarius 24SMB and Streptococcus oralis 89a administered as nasal spray in the treatment of recurrent upper respiratory tract infections in children	not FMT
Andaloro 2019	Bacteriotherapy with Streptococcus salivarius 24SMB and Streptococcus oralis 89a oral spray for children with recurrent streptococcal pharyngotonsillitis: a randomized placebo-controlled clinical study	not FMT
Feehley 2019	Healthy infants harbor intestinal bacteria that protect against food allergy	not FMT
Reeves 2018	Fibroblast gene expression following asthmatic bronchial epithelial cell conditioning correlates with epithelial donor lung function and exacerbation history	not FMT
Zhu 2018	Long noncoding RNA TUG1 promotes cardiac fibroblast transformation to myofibroblasts via miR-29c in chronic hypoxia	not FMT
Montassier 2018	CLOUD: a non-parametric detection test for microbiome outliers	not FMT
Hochman 2018	Immunoassay helps limit overdiagnosis of Clostridium difficile infection	not FMT
Bellussi 2018	Preventive nasal bacteriotherapy for the treatment of upper respiratory tract infections and sleep disordered breathing in children	not FMT
James 2018	Deficient Follistatin-like 3 Secretion by Asthmatic Airway Epithelium Impairs Fibroblast Regulation and Fibroblast-to-Myofibroblast Transition	not FMT
Tarantino 2018	Bacteriotherapy for preventing recurrent upper respiratory infections in children: a real-world experience	not FMT
Willyard 2018	Squeaky clean mice could be ruining research	not FMT
Jiang 2018	Comparison of two different combined test strips with fluorescent microspheres or colored microspheres as tracers for rotavirus and adenovirus detection	not FMT
Metwally 2018	MetaLonDA: a flexible R package for identifying time intervals of differentially abundant features in metagenomic longitudinal studies	not FMT
Gonzalo-skok 2017	AGE DIFFERENCES IN MEASURES OF FUNCTIONAL MOVEMENT AND PERFORMANCE IN HIGHLY YOUTH BASKETBALL PLAYERS	not FMT

Fischer 2017	Protein Malnutrition Alters Tryptophan and Angiotensin-Converting Enzyme 2 Homeostasis and Adaptive Immune Responses in Human Rotavirus-Infected Gnotobiotic Pigs with Human Infant Fecal Microbiota Transplant	not FMT
La Mantia 2017	Bacteriotherapy with Streptococcus salivarius 24SMB and Streptococcus oralis 89a nasal spray for preventing recurrent acute otitis media in children: a real-life clinical experience	not FMT
Ekmekciu 2017	The Probiotic Compound VSL#3 Modulates Mucosal, Peripheral, and Systemic Immunity Following Murine Broad-Spectrum Antibiotic Treatment	not FMT
Vemuri 2017	Therapeutic interventions for gut dysbiosis and related disorders in the elderly: antibiotics, probiotics or faecal microbiota transplantation?	not FMT
Huang 2017	Mutations in Interleukin-10 Receptor and Clinical Phenotypes in Patients with Very Early Onset Inflammatory Bowel Disease: A Chinese VEO-IBD Collaboration Group Survey	not FMT
Knoll 2017	Gut microbiota differs between children with Inflammatory Bowel Disease and healthy siblings in taxonomic and functional composition: a metagenomic analysis	not FMT
Isuiguro 2017	Biochemical analysis of intraplacental choriocarcinoma and fetomaternal transfusion	not FMT
Celerier 2017	Results of VSB implantation at the short process of the incus in children with ear atresia	not FMT
Yousuf 2017	Effect of Freeze Dried Powdered Probiotics on Gingival Status and Plaque Inhibition: A Randomized, Double-blind, Parallel Study	not FMT
Kuperman 2016	Antibiotic use during pregnancy: how bad is it?	not FMT
Shin 2016	Clostridium difficile Infection	not FMT
Fong 2016	A Novel Balance Training Program for Children With Developmental Coordination Disorder: A Randomized Controlled Trial	not FMT
Rodriguez 2016	Probiotic Compared with Standard Milk for High-caries Children: A Cluster Randomized Trial	not FMT
Fong 2016	Task-Specific Balance Training Improves the Sensory Organisation of Balance Control in Children with Developmental Coordination Disorder: A Randomised Controlled Trial	not FMT
Busch 2016	Comparison of Alternative Coupling Methods of the Vibrant Soundbridge Floating Mass Transducer	not FMT
Thomas 2015	Probiotics: a proactive approach to health. A symposium report	not FMT
Santagati 2015	Colonization, safety, and tolerability study of the Streptococcus salivarius 24SMBc nasal spray for its application in upper respiratory tract infections	not FMT
Patel 2015	New approaches for bacteriotherapy: prebiotics, new-generation probiotics, and synbiotics	not FMT
Franscini 2015	Pediatric reference intervals for plasma free and total metanephrines established with a parametric approach: relevance to the diagnosis of neuroblastoma	not FMT
Reeves 2015	Fibroblast-myofibroblast transition is differentially regulated by bronchial epithelial cells from asthmatic children	not FMT
Saraiva 2015	Epidemiology of infectious meningitis in the State of Amazonas, Brazil	not FMT
Kronman 2014	Intestinal decontamination of multidrug-resistant Klebsiella pneumoniae after recurrent infections in an immunocompromised host	not FMT
Tian 2014	Isolation and gut microbiota modulation of antibiotic-resistant probiotics from human feces	not FMT
Plontke 2014	Individual computer-assisted 3D planning for surgical placement of a new bone conduction hearing device	not FMT
Martins 2014	Clinical and virological descriptive study in the 2011 outbreak of dengue in the Amazonas, Brazil	not FMT
Sargsyan 2014	Hearing rehabilitation with single-stage bilateral vibroplasty in a child with Franceschetti syndrome	not FMT
Sammons 2013	Diagnosis and Management of Clostridium difficile Infection by Pediatric Infectious Diseases Physicians	not FMT

Zihler 2013	Novel Polyfermentor intestinal model (PolyFermS) for controlled ecological studies: validation and effect of pH	not FMT
Schwab 2013	Do we really need a Coupler for the round window application of an AMEI?	not FMT
Claud 2013	Bacterial community structure and functional contributions to emergence of health or necrotizing enterocolitis in preterm infants	not FMT
Madu 2013	Massive idiopathic feto-maternal transfusion associated with dilatation of umbilical vein: case report and review of literature	not FMT
Ciurea 2012	Fludarabine, melphalan, thiotepa and anti-thymocyte globulin conditioning for unrelated cord blood transplant	not FMT
Zernotti 2012	Middle ear implants: functional gain in mixed hearing loss	not FMT
Twetman 2012	Are we ready for caries prevention through bacteriotherapy?	not FMT
Beleites 2011	Experience with vibroplasty couplers at the stapes head and footplate	not FMT
Floch 2011	Recommendations for probiotic use-2011 update	not FMT
Mandala 2011	Treatment of the atretic ear with round window vibrant soundbridge implantation in infants and children: electrocochleography and audiologic outcomes	not FMT
Reix 2010	Bone mineral and body composition alterations in paediatric cystic fibrosis patients	not FMT
Keifer 2010	Combined aesthetic and functional reconstruction of ear malformations	not FMT
Stamatova 2009	Probiotics: health benefits in the mouth	not FMT
Skovbjerg 2009	Spray bacteriotherapy decreases middle ear fluid in children with secretory otitis media	not FMT
Caramina 2008	Metchnikoff and the centenary of probiotics: an update of their use in gastroenteric pathology during the age of development	not FMT
Roessner 2008	Color perception deficits in co-existing attention-deficit/hyperactivity disorder and chronic tic disorders	not FMT
Braillon 2007	Bone mineral content and body composition in overweight children and adolescents	not FMT
Dobalian 2006	Advance care planning documents in nursing facilities: results from a nationally representative survey	not FMT
Banaschewski 2006	Colour perception in ADHD	not FMT
Borborema-Santos 2006	Oral focal epithelial hyperplasia: report of five cases	not FMT
De Figueiredo 2004	Exanthematous diseases and the first epidemic of dengue to occur in Manaus, Amazonas State, Brazil, during 1998-1999	not FMT
Todt 2004	MRI scanning and incus fixation in vibrant soundbridge implantation	not FMT
Shaw 2004	The effect of initial stimulus type for visual reinforcement audiometry	not FMT
Roberts 2004	Prenatal stress, moderate fetal alcohol, and dopamine system function in rhesus monkeys	not FMT
Walls 2003	Bacteriocin-like inhibitory substance (BLIS) production by the normal flora of the nasopharynx: potential to protect against otitis media?	not FMT
Brigidi 2003	PCR detection of Bifidobacterium strains and Streptococcus thermophilus in feces of human subjects after oral bacteriotherapy and yogurt consumption	not FMT
Rosenfeldt 2003	Effect of probiotic Lactobacillus strains in children with atopic dermatitis	not FMT
Hatayama 2003	Evaluation of hemangioma by positron emission tomography: role in a multimodality approach	not FMT
Rosenfeldt 2002	Effect of probiotic Lactobacillus strains in young children hospitalized with acute diarrhea	not FMT

Eberling 2002	No effect of age and estrogen on aromatic L- amino acid decarboxylase activity in rhesus monkey brain	not FMT
Helin 2002	No effect of oral treatment with an intestinal bacterial strain, Lactobacillus rhamnosus (ATCC 53103), on birch-pollen allergy: a placebo-controlled double-blind study	not FMT
Watanabe 2000	PET imaging of musculoskeletal tumours with fluorine-18 alpha-methyltyrosine: comparison with fluorine-18 fluorodeoxyglucose PET	not FMT
Kirjavainen 1999	Healthy gut microflora and allergy: factors influencing development of the microbiota	not FMT
Inoue 1999	18F alpha-methyl tyrosine PET studies in patients with brain tumors	not FMT
Ullman 1998	Yogurt as oral bacteriotherapy for diarrhea: back to the future?	not FMT
Shornikova 1997	Bacteriotherapy with Lactobacillus reuteri in rotavirus gastroenteritis	not FMT
Malin 1996	Increased bacterial urease activity in faeces in juvenile chronic arthritis: evidence of altered intestinal microflora?	not FMT
Malin 1996	Promotion of IgA immune response in patients with Crohn's disease by oral bacteriotherapy with Lactobacillus GG	not FMT
Slabospitskaia 1995	A new preparation of biosporin and its effect on the intestinal microflora in dysbacterioses in newborn infants	not FMT
Isolauri 1994	Oral bacteriotherapy for viral gastroenteritis	not FMT
Gonzalez 1994	Biotherapeutic role of fermented milk	not FMT
Balli 1992	High-dose oral bacteria-therapy for chronic non-specific diarrhea of infancy	not FMT
Vanderby 1990	Acute modification of biomechanical properties of the bone-ligament insertion to rat limb unweighting	not FMT
Tvede 1990	Bacteriotherapy for Clostridium difficile diarrhoea	not FMT
Benoni 1984	Antibiotic administration and oral bacterial therapy in infants	not FMT
Zoppi 1982	Oral bacteriotherapy in clinical practice. II. The use of different preparations in the treatment of acute diarrhoea	not FMT
Zoppi 1982	The use of different preparations in infants treated with antibiotics	not FMT
Elkeles 1967	Bacteriotherapy and stimulation therapy inspite, instead and with chemotherapy]	not FMT
Xi 2021	Depicting the composition of gut microbiota in children with tic disorders: an exploratory study	not FMT
Feng 2021	Fecal microbiota from children with vitamin A deficiency impair colonic barrier function in germ-free mice: The possible role of alterative bile acid metabolites	not FMT
Feng 2022	Microorganisms in the reproductive system and probiotic's regulatory effects on reproductive health	Not FMT
Gedam 2022	Efficacy of Probiotic, Chlorhexidine, and Sodium Fluoride Mouthrinses on Mutans Streptococci in 8- to 12-Year-Old Children: A Crossover Randomized Trial	Not FMT
Cardile, S; Pietrobattista, A; Del Chierico, F; Vernocchi, P; Russo, A; Bracaglia, G; Bernaschi, P; Pansani, L; Argentieri, M; Putignani, L; Torre, G	Gut microbiota profiling in an infant colonized by multiresistent germ candidate to liver transplantation	not FMT

Bekker 2019	Dynamics of the Gut Microbiota in Children Receiving Selective or Total Gut Decontamination Treatment during Hematopoietic Stem Cell Transplantation	not FMT
Del Chierico 2018	Liver Transplantation and Gut Microbiota Profiling in a Child Colonized by a Multi-Drug Resistant Klebsiella pneumoniae: A New Approach to Move from Antibiotic to "Eubiotic" Control of Microbial Resistance	not FMT
Simms-Waldrip 2017	Antibiotic-Induced Depletion of Anti-inflammatory Clostridia Is Associated with the Development of Graft-versus-Host Disease in Pediatric Stem Cell Transplantation Patients	not FMT
Taggart 2014	An Overview of the Microbiome and the Effects of Antibiotics	not FMT
Song 2021	Exploration of the relationship between intestinal flora changes and gut acute graft-versus-host disease after hematopoietic stem cell transplantation	not FMT
Sarbagili-Shabat 2021	Novel UC Exclusion Diet and Antibiotics for Treatment of Mild to Moderate Pediatric Ulcerative Colitis: A Prospective Open-Label Pilot Study	not FMT
Talathi 2021	Scheduled Empiric Antibiotics May Alter the Gut Microbiome and Nutrition Outcomes in Pediatric Intestinal Failure	not FMT
Coker 2021	Infant Feeding Alters the Longitudinal Impact of Birth Mode on the Development of the Gut Microbiota in the First Year of Life	not FMT
Gong 2021	Effects of antibiotic treatment and probiotics on the gut microbiome of 40 infants delivered before term by cesarean section analysed by using 16s rRNA quantitative polymerase chain reaction sequencing	not FMT
Alexander 2021	Neutrophil functional profiling and cytokine augmentation for patients with multiple recurrent infections: A case study	not FMT
Kim 2020	Delayed Establishment of Gut Microbiota in Infants Delivered by Cesarean Section	not FMT
Xie 2019	Impaired Chylomicron Assembly Modifies Hepatic Metabolism Through Bile Acid-Dependent and Transmissible Microbial Adaptations	not FMT
Zheng 2019	Dietary Therapy in Conjunction with Immunosuppression to Treat Gastrointestinal Graft-versus-host Disease (GVHD)	not FMT
Bunyavanich 2019	Food allergy: could the gut microbiota hold the key?	not FMT
Contijoch 2019	Gut microbiota density influences host physiology and is shaped by host and microbial factors	not FMT
Kang 2020	Distinct Fecal and Plasma Metabolites in Children with Autism Spectrum Disorders and Their Modulation after Microbiota Transfer Therapy	Not reporting ASD outcomes after FMT
NCT03582969,	Capsulized Fecal Microbiota Transplantation in Pediatric Ulcerative Colitis Patients	Ongoing Study
NCT05202990,	Oral Fecal Microbiota Transplantation in Pediatric Ulcerative Colitis	Ongoing Study
Zeevenhooven 2020	Protocol for a pilot randomised, double-blind, placebo-controlled trial for assessing the feasibility and efficacy of faecal microbiota transplantation in adolescents with refractory irritable bowel syndrome: FAIS Trial	Protocol
Leong 2019	Protocol for the Gut Bugs Trial: a randomised double-blind placebo-controlled trial of gut microbiome transfer for the treatment of obesity in adolescents	Protocol
Pai 2019	Protocol for a double-blind, randomised, placebo-controlled pilot study for assessing the feasibility and efficacy of faecal microbiota transplant in a paediatric Crohn's disease population: PediCRaFT Trial	Protocol
Pai 2017	Protocol for a randomised, placebo-controlled pilot study for assessing feasibility and efficacy of faecal microbiota transplantation in a paediatric ulcerative colitis population: PediFETCh trial	protocol
Helve 2021	Protocol for oral transplantation of maternal fecal microbiota to newborn infants born by cesarean section	Protocol
Fang 2018	Protocol for Fecal Microbiota Transplantation in Inflammatory Bowel Disease: A Systematic Review and Meta-Analysis	Protocol
Kellermayer 2022	Fecal Microbiota Transplantation Commonly Failed in Children With Co-Morbidities	Review
Frye 2015	Approaches to studying and manipulating the enteric microbiome to improve autism symptoms	review

Marra 2015	Controversies Around Epidemiology, Diagnosis and Treatment of Clostridium difficile Infection	review
Vandenplas 2015	Fecal Microbiota Transplantation: Just a Fancy Trend?	review
Esposito 2015	Treatment of Clostridium difficile infection in pediatric patients	review
Russell 2015	Too early to determine whether fecal microbiota transplant has therapeutic promise for Ulcerative Colitis?	review
Kassam 2014	Review of the emerging treatment of Clostridium difficile infection with fecal microbiota transplantation and insights into future challenges	review
Walia 2014	Fecal microbiota transplantation in the treatment of refractory Clostridium difficile infection in children: an update	review
Dickinson 2014	Infectious diarrhea: an overview	review
Samuel 2014	What nurses need to know about fecal microbiota transplantation: education, assessment, and care for children and young adults	review
Kellermayer 2013	Prospects and challenges for intestinal microbiome therapy in pediatric gastrointestinal disorders	review
Davidovics 2013	Fecal transplantation: re-discovering the value of stool	review
Kahn 2012	Fecal bacteriotherapy for ulcerative colitis: patients are ready, are we?	review
Brandt 2011	Fecal microbiota transplantation for recurrent clostridium difficile infection	review
Slattery 2016	The Significance of the Enteric Microbiome on the Development of Childhood Disease: A Review of Prebiotic and Probiotic Therapies in Disorders of Childhood	review
McFarland 2016	Comparison of pediatric and adult antibiotic-associated diarrhea and Clostridium difficile infections	review
Warner 2018	he contribution of the gut microbiome to neurodevelopment and neuropsychiatric disorders	review
Fischer 2019	Recent research on fecal microbiota transplantation in inflammatory bowel disease patients	review
No authors 2014	CRITICAL VIEWS IN GASTROENTEROLOGY & HEPATOLOGY: Fecal Microbiota Transplantation: Where Is It Leading?	review
No authors 2014	CRITICAL VIEWS IN GASTROENTEROLOGY & HEPATOLOGY: Fecal Microbiota Transplantation: Where Is It Leading?	review
Gersten 2021	Fecal matter transplant and nursing care: Combating clostridioides difficile infections	review
Adams 2022	Community-acquired clostridioides difficile cases in children on the rise: Increase in pediatric cases of C difficile is linked to use of broad-spectrum antibiotics, recurrent infections, and a growth in highly virulent strains	review
Noor 2018	Clostridium difficile infection in children	review
Schutze 2013	Clostridium difficile infection in infants and children	review
Herman 2022	Could Candida Overgrowth Be Involved in the Pathophysiology of Autism?	review
Tu 2021	Treating autism spectrum disorder by intervening with gut microbiota	review
Zebrowska 2021	Future Directions in Reducing Gastrointestinal Disorders in Children With ASD Using Fecal Microbiota Transplantation	review
Adams 2019	Microbiota transplant therapy and autism: lessons for the clinic	review
Yang 2018	Targeting gut microbiome: A novel and potential therapy for autism	review
Jain 2021	Gut Microbiome: A Potential Modifiable Risk Factor in Biliary Atresia	review
Vasilescu 2022	Gut Dysbiosis and Clostridioides difficile Infection in Neonates and Adults	review

Bernard 2021	Fecal Microbiota Transplantation and Microbial Therapeutics for the Treatment of Clostridioides difficile Infection in Pediatric Patients	review
Conrad 2021	Clostridioides difficile Infection in Pediatric Inflammatory Bowel Disease: A Clinician's Dilemma	review
Kociolek 2021	Recent advances in Clostridioides difficile infection epidemiology, diagnosis and treatment in children	review
Cotter 2019	An Infectious Diseases Perspective on Fecal Microbiota Transplantation for Clostridioides difficile Infection in Children	review
Chen 2022	Current and future applications of fecal microbiota transplantation for children	review
Parnell 2021	Pediatric Fecal Microbiota Transplantation in Recurrent Clostridioides Difficile	review
Pane 2021	Clinical Parasitology and Parasitome Maps as Old and New Tools to Improve Clinical Microbiomics	review
MacLellan 2021	Age-Matching in Pediatric Fecal Matter Transplants	review
Masetti 2020	The gut microbiome in pediatric patients undergoing allogeneic hematopoietic stem cell transplantation	review
Eindor-Abarbanel 2021	Therapeutic Advances in Gut Microbiome Modulation in Patients with Inflammatory Bowel Disease from Pediatrics to Adulthood	review
Yang 2021	Advances in the Relationships Between Cow's Milk Protein Allergy and Gut Microbiota in Infants	review
Cetkin 2021	Adjunctive Treatment of Pediatric Adenoidal Hypertrophy: A Review	review
Popov 2021	Microbiota-Immune Interactions in Ulcerative Colitis and Colitis Associated Cancer and Emerging Microbiota-Based Therapies	review
Alam 2022	Manipulating Microbiota to Treat Atopic Dermatitis: Functions and Therapies	review
Joachim 2022	Fecal Microbiota Transfer (FMT) in Children and Adolescents – Review and statement by the GPGE microbiome working group	review
Shehata 2022	Microbiomes in physiology: insights into 21st-century global medical challenges	review
Krutova 2022	How to: Clostridioides difficile infection in children	review
Li 2022	Targeting the Pulmonary Microbiota to Fight against Respiratory Diseases	review
Durovic 2021	Cutting edges in Clostridioides difficile infections	review
Oldenburg 2021	The Microbiome in Childhood Acute Lymphoblastic Leukemia	review
Zhu 2021	Prospects for clinical applications of butyrate-producing bacteria	review
Fianchi 2021	Nonalcoholic Fatty Liver Disease (NAFLD) as Model of Gut-Liver Axis Interaction: From Pathophysiology to Potential Target of Treatment for Personalized Therapy	review
Akutko 2021	Probiotics, Prebiotics and Synbiotics in Inflammatory Bowel Diseases	review
Albuhairi 2021	Biologics and Novel Therapies for Food Allergy	review
Cuna 2021	Dynamics of the preterm gut microbiome in health and disease	review
Rachid 2021	The microbial origins of food allergy	review
Gomaa 2020	Human gut microbiota/microbiome in health and diseases: a review	review
Ciprandi 2020	Local Bacteriotherapy - a promising preventive tool in recurrent respiratory infections	review
Giles 2020	Microbiome in health and disease	review

Thom 2020	Immune Modulatory Treatments for Autism Spectrum Disorder	review
Lim 2020	Engineering the Gut Microbiome for Treatment of Obesity: A Review of Current Understanding and Progress	review
Schwartz 2020	Understanding the impact of antibiotic perturbation on the human microbiome	review
Park 2020	Increasing incidence of inflammatory bowel disease in children and adolescents: significance of environmental factors	review
Stephen-Victor 2020	Dietary and Microbial Determinants in Food Allergy	review
Yaghoubi 2020	p28 Bacterial Peptide, as an Anticancer Agent	review
Bajinka 2020	Extrinsic factors influencing gut microbes, the immediate consequences and restoring eubiosis	review
Nance 2020	The Role of the Microbiome in Food Allergy: A Review	review
Gnocchi 2020	Updated Management Guidelines for Clostridioides difficile in Paediatrics	review
Ebrahimzadeh Leylabadlo 2020	Non-alcoholic fatty liver diseases: from role of gut microbiota to microbial-based therapies	review
Southwell 2020	Treatment of childhood constipation: a synthesis of systematic reviews and meta-analyses	review
Olesen 2020	Global disparities in faecal microbiota transplantation research	review
Mounsey 2020	Clostridioides difficile Infection: Update on Management	review
Harkins 2020	Manipulating the Human Microbiome to Manage Disease	review
Pironi 2019	Management of the Patient with Chronic Intestinal Pseudo-Obstruction and Intestinal Failure	review
Ersoz 2019	The Role of Gut Microbiota in Autism Spectrum Disorder	review
Gurram 2019	Fecal microbiota transplantation in children: current concepts	review
Kujiper 2019	Treatment of (recurrent) Clostridioides difficile Infections in Children and Adults	review
Wardill 2019	Adjunctive fecal microbiota transplantation in supportive oncology: Emerging indications and considerations in immunocompromised patients	review
Campbell 2019	An Updated Review of Clostridium difficile Treatment in Pediatrics	review
Dalzell 2019	Paediatric inflammatory bowel disease: review with a focus on practice in low- to middle-income countries	review
Lauro 2019	Short bowel syndrome in children and adults: from rehabilitation to transplantation	review
Nobili 2019	Fighting Fatty Liver Diseases with Nutritional Interventions, Probiotics, Symbiotics, and Fecal Microbiota Transplantation (FMT)	review
Gupta 2018	Diagnosis and Treatment of Clostridium difficile Infection	review
Huang 2018	Microbial treatment in chronic constipation	review
Schaffler 2018	Clostridium difficile - From Colonization to Infection	review
Quraishi 2017	Faecal transplantation for IBD management-pitfalls and promises	review
Clayton 2017	Recent Issues in Pediatric Clostridium difficile Infection	review
Walker 2017	The importance of appropriate initial bacterial colonization of the intestine in newborn, child, and adult health	review

Guariso 2017	Treating children with inflammatory bowel disease: Current and new perspectives	review
Chen 2017	Fecal microbiota transplantation for recurrent clostridium difficile infection in children	review
Al Khodor 2017	Gut microbiome and kidney disease: a bidirectional relationship	review
Martin 2017	Gut-Brain Axis and Behavior	review
Wang 2016	Fecal microbial transplant for the treatment of pediatric inflammatory bowel disease	review
Qiao 2016	Therapeutic modulation of gut microbiota in inflammatory bowel disease: More questions to be answered	review
Grady 2016	Microbial therapeutic interventions	review
Li 2016	The gut microbiota: A treasure for human health	review
Hourigan 2016	Fecal microbiota transplantation in children: a brief review	review
Vieira 2016	New insights into therapeutic strategies for gut microbiota modulation in inflammatory diseases	review
Landman 2016	[Gut microbiota: Description, role and pathophysiologic implications]	review
Grigorescu 2016	IMPLICATION OF GUT MICROBIOTA IN DIABETES MELLITUS AND OBESITY	review
Fuentes 2016	How to Manipulate the Microbiota: Fecal Microbiota Transplantation	review
Dinh 2015	Current treatment and epidemiology of Clostridium difficile infections	review
Pawasarat 2021	Constipation in pediatrics: A clinical review	Review
Nicholson 2021	Current Challenges in Fecal Microbiota Transplantation for Clostridioides difficile Infection in Children	Review
Yang 2020	Effects of gut microbial-based treatments on gut microbiota, behavioral symptoms, and gastrointestinal symptoms in children with autism spectrum disorder: A systematic review	Systematic review
Martinez-Gonzalez 2020	Prebiotics, probiotics and fecal microbiota transplantation in autism: A systematic review	Systematic review
Sharpton 2019	Gut microbiome-targeted therapies in nonalcoholic fatty liver disease: a systematic review, meta-analysis, and meta-regression	Systematic review
Iqbal 2018	Safety and efficacy of encapsulated fecal microbiota transplantation for recurrent Clostridium difficile infection: a systematic review	Systematic review
Sha 2014	Systematic review: faecal microbiota transplantation therapy for digestive and nondigestive disorders in adults and children	Systematic review
Tariq 2021	Outcomes of Fecal Microbiota Transplantation for C. difficile Infection in Inflammatory Bowel Disease: A Systematic Review and Meta-analysis	Systematic review and meta analysis
Yang 2020	Effects of gut microbial-based treatments on gut microbiota, behavioral symptoms, and gastrointestinal symptoms in children with autism spectrum disorder: A systematic review	Systematic review and meta analysis
